# Insights from the Niger Delta Region, Nigeria on the impacts of urban pollution on the functional organisation of Afrotropical macroinvertebrates

**DOI:** 10.1038/s41598-022-26659-0

**Published:** 2022-12-29

**Authors:** Augustine Ovie Edegbene, Frank Chukwuzuoke Akamagwuna

**Affiliations:** 1 Institute for Water Research, Rhodes University, Makhanda (Grahamstown), 6140 South Africa; 2 Department of Biological Sciences, Federal University of Health Sciences, Otukpo, Nigeria

**Keywords:** Ecology, Ecosystem ecology, Freshwater ecology, Urban ecology

## Abstract

Anthropogenic activities, including urbanisation and industrialisation threaten stream ecological integrity, ecosystem community structure and ecosystem functioning of rivers and streams worldwide. However, developing sustainable monitoring strategies for ecological health remains a critical challenge in Africa. We examined the effects of urban disturbance on macroinvertebrate Functional Feeding Groups in selected streams in the Niger Delta Region of Nigeria. We sampled 11 sites between 2008 and 2012 and grouped into three site groups (Site groups 1 > 2 > 3). The groups represent an increasing gradient of urban pollution. Our result showed that urban-induced disturbances affected physicochemical variables in the study area (PERMANOVA; *p* < 0.05), with nutrients NO_2_-N, PO_4_-P, and electrical conductivity being significantly higher in impacted Site group 3 (ANOVA, *p* < 0.05). Predators and gatherers were the most dominant Functional Feeding Group recorded in the study area, while shredders were the least abundant macroinvertebrate Functional Feeding Groups. The multivariate RLQ analysis revealed that shredders, predators, and scrapers were tolerant of urban pollution, whereas gatherers were sensitive to increasing urban pollution. Overall, macroinvertebrates Functional Feeding Groups responded differentially to urban pollution in the Niger Delta Region. Identifying pollution indicator Functional Feeding Groups is seen as an important step towards developing a reliable, low-cost tool for riverine monitoring of urban pollution effects in Africa.

## Introduction

Freshwater ecosystems provide desired and valuable services to humans and animals, but they are threatened by human activities associated with urban developmental changes^1,2^. Urbanisation, economic and industrial development threaten stream ecological integrity and ecosystem functioning of rivers and streams worldwide^3,4^. Urban development exposes streams and rivers to sedimentation, nutrient enrichment, pesticides, and other toxic pollutants^5^, affecting the ecological status and ecosystem functioning^6,7^.

In developing countries such as Nigeria, the problem of urban pollution of riverine systems is exacerbated by rapid population growth and rural–urban migration^8–11^, and most times leading to nutrient and sediment input^12,13^, flow- and temperature-regime alterations^12,14^, degradation of riparian and in-stream habitats^15^ and toxicant inputs, and ultimately alteration of physicochemistry of the affected systems. A significant consequence of the physicochemical alterations caused by urban pollution includes changes in the biological community structure (e.g., macroinvertebrates) and ecological processes, such as food webs of impacted rivers and streams^2,10^. Temporal heterogeneity of hydrological regimes is also a fundamental process in shaping changes associated with urban pollution, and their impact on the structure and function of riverine macroinvertebrates^16^. For example, seasonal changes influence the hydrology, water temperature, flow and nutrients regimes in most rivers^17^. Thus, seasons can either exacerbate or ameliorate the effects of urban mediated environmental changes on biological community structure. Macroinvertebrates are one of the most diverse biota in river and stream habitats^18^, and they play an essential role in the energy flow of freshwater ecosystems^19,20^. They represent the main sources of primary productivity through leaf litter decomposition^21,22^. For example, macroinvertebrates are estimated to process 20–73% of riparian leaf-litter inputs to headwater streams^21^. Invertebrate predators are also seen to control the numbers, locations, and sizes of their prey in streams^23^. As a result, their structural and functional metrics are the most widely used in bioindication of ecological health due to their wide sensitivity to environmental disturbance, broad diversity, and abundance in riverine systems^24,25^. Macroinvertebrates’ functional and structural assemblage can respond differentially to environmental perturbation from urbanisation and its associated ecological alteration. For example, macroinvertebrate shredders and gatherers decrease in abundance and diversities in response to riparian habitat alteration resulting from urban advancement^26^. Changes in nutrient load and temperature regime from urban development have been shown to significantly alter sensitive macroinvertebrate groups (e.g., Ephemeroptera Plecoptera Trichoptera (EPT) communities and basal reassures for the food web in streams^27^. Seasonal changes in macroinvertebrate communities hugely rely on functional traits such as life-history and functional feeding habits^28,29^. Specialist macroinvertebrate feeders such as scrapers, shredders and predators are more abundant during the wet season due to maximal resource levels^30^, whereas generalist feeding collector-gatherers tend to increase during the wet and dry seasons^31^.

Because macroinvertebrates exhibit varying behavioural and physiological mechanisms to obtain food and habitat utilisation^32,33^, they have been classified into distinct functional feeding groups (FFGs—predators, shredders, gatherers, filterers, scrapers and grazers) and used for biomonitoring programmes^29,34^. The classification method is helpful in that a specific number of macroinvertebrate individuals from a community can be studied collectively based on their behaviour and adaptations for feeding in the stream environment^29,32^. Thus, the preferential requirements for food and habitat, which vary along an environmental gradient, enable us to employ macroinvertebrate FFGs as indicators of ecological status and ecosystem functioning^35–37^. Shredders have been shown to be less abundant in highly urbanised streams, whereas filter-feeders and collectors increases^9,10^. As a result, researchers have used macroinvertebrate FFGs to monitor environmental disturbance, including urban pollution in water bodies (e.g.^38–40^).

Despite the applicability of macroinvertebrates FFGs for biomonitoring aquatic ecosystems, our knowledge of the response of FFGs to environmental disturbance, including urban pollution, are limited to other regions of the world. Lack of requisite taxonomic expertise and resources are notable challenges hindering the use of macroinvertebrates FFGs in developing biomonitoring programmes in the Afrotropical region, unlike the case in the developed regions^31,41^. Therefore, exploring the use of macroinvertebrates FFGs in assessing streams health in the present study is timely, as it has been reported in several studies that macroinvertebrate FFGs responds differentially to anthropogenic influences^39,42^, and can be used to indicate ecological health and ecosystem functioning of streams and rivers impacted by urban pollution when resources are scarce.

The Niger Delta area of Nigeria was home to numerous forested streams and rivers. Most of these streams and rivers are now draining urban catchment due to increased urban advancement to accommodate the teeming pollution growth. As a result, previous regional studies have shown that the loss of forested riparian catchments have grave consequences on streams’ abiotic and biotic components^26^. Further, the area hosts numerous oil exploration activities^43^, contributing to water quality deterioration and biodiversity loss. Consequently, communities in the region face limited access to quality water supply, with severe implications on human health, economy and society^44^.

Utilising macroinvertebrate FFGs to estimate ecological quality is seen as one of the most useful and less expensive techniques appropriate for the region. Presently, Nigeria doesn’t have an established biomonitoring programme to monitor ecological health and manage water bodies. Therefore, exploring the potential use of macroinvertebrate FFGs for biomonitoring in the region can provide an effective and low-cost tool for the long-term monitoring of water bodies in the Niger Delta. We hypothesised that FFGs could be explored and applied in assessing the ecological health of streams in the region impacted by urban pollution and that tolerant and sensitive indicators FFGs of urban pollution can be identified. For this, we (i) investigated the spatial and seasonal changes in the relative abundance of FFGs along urban pollution gradients, (ii) used the multivariate RLQ analysis to discern the distribution patterns of FFGs in relation to physicochemical variables.

## Materials and methods

### Study area and sampling sites

**Figure 1:**
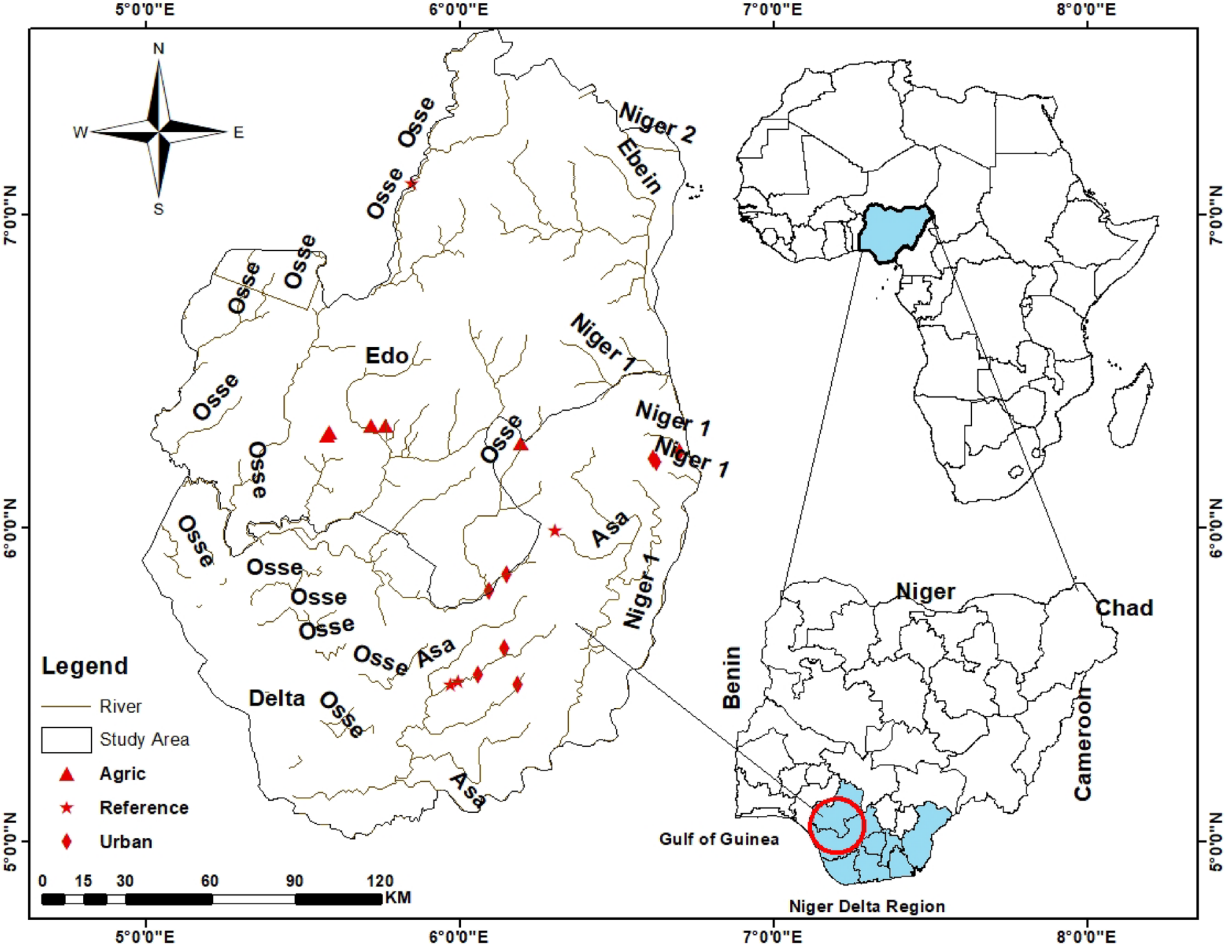
Location of urban streams sampling sites in Edo and Delta States within the Niger Delta Region of Nigeria.

We studied 11 first- to fourth-order streams in the Delta and Edo States administrative boundaries of the Niger Delta Region, Nigeria (Fig. 1). The rivers are situated at the interception of latitude 5° 50ʹ 40ʹʹ–7° 09ʹ 03ʹʹ N and longitude 5° 96ʹ 37ʹʹ–6° 71ʹ 10ʹʹ E, and include the following streams Adofi, Orogodo, Ethiope, Warri, Ogba, Obosh Oleri and Anwai. The area’s climate is mainly tropical and humid, with two distinct seasons, wet and dry. The mean annual rainfall ranges from 900 to 2200 mm, and temperature ranges from 25 to 35 °C, which varies greatly with the season^9^. The streams drain different land-use types with varying human population densities and pressures^9,26^. The region supports a wide range of subsistence inland fisheries and wood logging^45^, and is known for extensive oil exploration activities^46^.

Many driving forces related to urban activities have led to the contamination of urban rivers in Nigeria. The most notable driving forces in the Niger Delta Region include population density, use of public latrines, residential and informal settlements, industrial advancement, agriculture and oil exploration and power plants^43,47^. Some of the potentials urban pollutants in the selected urban rivers include heavy metals, pesticides, fertilizers and plastics wastes. Further, natural sources of pollution in the studied sites include urban storm-water return flow, increased sedimentation resulting from erosion and excessive growth of macrophytes resulting in decayed organic matters^9^. Sites were carefully selected to represent an increasing gradient of urban pollution. We selected sites in streams that drain the major cities in the Edo and Delta States which account of major urban activities in the area such as high population densities, agro-industrial, oil exploration and indiscriminate waste dumping sites (Fig. 1). Other factors such as site accessibility and availability of macroinvertebrate sampling habitats were also considered in selecting sites. All selected sites are freshwater ecosystems. We delineated study sites into three categories characterised by catchment or riparian land-use and reach-scale human influences, including forested, agricultural, mixed and urban. We used objective criteria and best professional judgment to select the least impacted sites as control (reference)^48,49^. Grouping the sites into three site categories helped to reduce site redundancy. We further used the cluster analysis based on physicochemical variables collected from the 11 sites across the eight streams sampled to classify the sites into site groups, confirming the initial site classification (Fig. S1, Supplementary Information). The three site groups formed an increasing gradient of urban pollution stress from the least impacted Site group 1 (Site 1 = Adofi, Site 2 = Oleri, and Site 3 = Anwai upstream, Site 4 = Anwai downstream and Site 9 = Ogba upstream site) > moderately impacted Site group 2 (Site 5 = Ethiope upstream, Site 6 = Ethiope downstream, Site 7 = Obosh and Site 8 = Ogba downstream) > the highly impacted Site group 3 (Site 10 = Orogodo and Site 11 = Warri River). These three site groups formed the basis for subsequent analyses in this study.

### Sampling

Physiochemical variables and macroinvertebrates were collected from the 11 sites monthly during the dry and wet seasons of 2008 and 2012. The dry season samples were collected during high wind and temperature of November to March months, whereas the wet season samples were collected during short light rainfalls of April to October. In total, 84 samples were collected during both seasons, 42 samples each collected during the dry and wet seasons during the sampling period. We measured water quality variables at each sampling event, including dissolved oxygen (DO), pH, water temperature, and electrical conductivity (EC), before collecting macroinvertebrates. Dissolved oxygen (DO) was measured using DO meter; model YSI 55, and EC, pH, and temperature were taken using the portable multi-meter analyser HANNA HI 9913001/1. Habitat variables, including water depth and water velocity (m/s), were taken with a calibrated rod (measured in meters) and timing a weighted cork for a distance of 10 m, respectively. We collected water samples from each site during each sampling event using 500 ml sample bottles to measure nutrients, including nitrate (NO_3_-N) and phosphate (PO_4_-P), and biological oxygen demand (BOD_5_), and they were analysed following standard method^50^. BOD_5_ is the difference between the concentration of dissolved oxygen (DO) in water sample taken instantaneously and concentration of DO that has been incubated for 5 days at 20 °C in the dark.

Macroinvertebrates were sampled using a D-frame kick net (30 × 30 cm aperture and 0.05 cm mesh size) sampling method^51^ in line with South African Scoring System version 5 (SASS5^51^). We kick sampled macroinvertebrates from three different biotopes (i.e., in-stream macroinvertebrate habitats) at each site and sampling event, including stones, sediments, and vegetation. Stone biotope is pebbles and cobbles (2–25 cm), boulders (> 25 cm) situated in riffles (stones in currents) and pools (stones out of current). Vegetation biotopes include marginal vegetation growing on the river edge, fringing into the river, and aquatic vegetation submerged in the main river channel. Sediment biotope was gravels (small stones usually less than 2 cm in diameter), sand and mud that are less than 2 mm and 0.06 mm, respectively. For stones, we sampled stones in-current and out-of-current for 3 min, and sediments (gravel, sand, and mud) were sampled for 1 min, whereas 1 m^2^ of marginal and aquatic vegetation, respectively, were sampled for vegetation. Large substrate types such as boulders and cobbles were disturbed by hand and washed into the net. We grouped the three subsamples into a single pooled composite sample for each biotope per site on every sampling event. Each macroinvertebrate sample collected from each biotope were preserved in 70% ethanol and transported to the laboratory for analysis.

### Laboratory analysis

Water sampled for nutrients: nitrate (NO_3_-N), phosphate (PO_4_-P), and BOD_5_ were analysed within 24 h of collection, following standard procedure^50^. Macroinvertebrates samples were sorted by hand-picking from sediment and identified to families using taxonomic keys by Refs.^52–54^. Each macroinvertebrate taxon was placed into one of six FFG classes, including filter-feeders, gatherers, scrapers, grazers, predators, and shredders using the literature^54–58^. The FFG classification was done at family level taxonomic classification. Previous studies have demonstrated that family level classification of FFGs is sufficient for the detection of environmental changes^59,60^.

### Data analysis

The canonical analysis of principal coordinates (CAP^61^) based on Euclidean distance was used to investigate the differences in physicochemical variables across the site categories during the dry and wet seasons. We used two-way permutational multivariate analysis of variance, PERMANOVA to test the changes in physiochemical variables between the land use categories and seasons. Two-way analysis of variance tested the differences in the relative abundance of macroinvertebrate FFGs across the three site categories for the dry and wet seasons. The Tukey post hoc test revealed the site groups that differed significantly. Before conducting ANOVA, we used Shapiro Wilks and Levene’s tests to assess the assumption of homogeneity and equality of variance.

The relationships between physicochemical variables associated with urban pollution and macroinvertebrate FFGs collected in the Niger Delta Region were examined using the multivariate RLQ analysis. The R-environmental variable dataset, L-abundance dataset and Q-trait dataset analysis is a three-step multivariate ordination method routinely used to link species traits (e.g., FFGs and life-history traits) to environmental variables using the species abundance data (see^62^). The Q trait dataset was obtained by assigning FFGs to macroinvertebrate taxa using a binary coding approach^63^ (Supplementary Data, Table S1). The first ordination, correspondence analysis, CA is conducted on the taxa data set, i.e., L-table, followed by a principal component analysis, PCA on the environmental variables dataset, i.e., R-table. The PCA ordination allows linking the taxa dataset to the environmental variable’s dataset using the CA’s sample output as row weights. The third ordination, Hill-smith analysis (HS) is conducted on traits (the macroinvertebrate FFGs dataset), which allow the linking of the taxa dataset to the trait (FFGs) dataset using the output results of the CA’s taxon scores as row weights. The final step of the RLQ procedure simultaneously performs ordinations on the three separate ordinations. The trait dataset (Q-table) was the binary coded macroinvertebrate FFGs dataset for the RLQ analysis. The taxa abundance dataset, L-table, used the macroinvertebrate abundance dataset from the 42 sampling exercises each for both dry and wet season in the 11 sites in the five years study period. Same was done for the environmental variables dataset (physiochemical variables), i.e., R-table. The FFGs matrixes were derived by binary coding the taxa abundance data for the Q-table as we stated above.

The statistical significance of the RLQ procedure was examined with the Monte-Carlo test. We used the Model 6 method advocated by Dray et al.^64^ that produces two p-values (i.e., Models 2 and 4) for the significance test. 999 permutation was used for RLQ analysis based on the false discovery rate method for p-value adjustment. Model 2 tests the null hypothesis that the environment did not influence species composition, whereas Model 4 tests that traits (i.e., FFGs) did not affect species distribution patterns. All ordination analyses, including the RLQ and associated procedures, were conducted in R software using ADE-4^65^. ANOVA, Shapiro Wilks and Levene’s tests were conducted in Statistica, and PERMANOVA in Primer 6+ PERMANOVA+.

## Results

### Physicochemical variables

**Figure 2:**
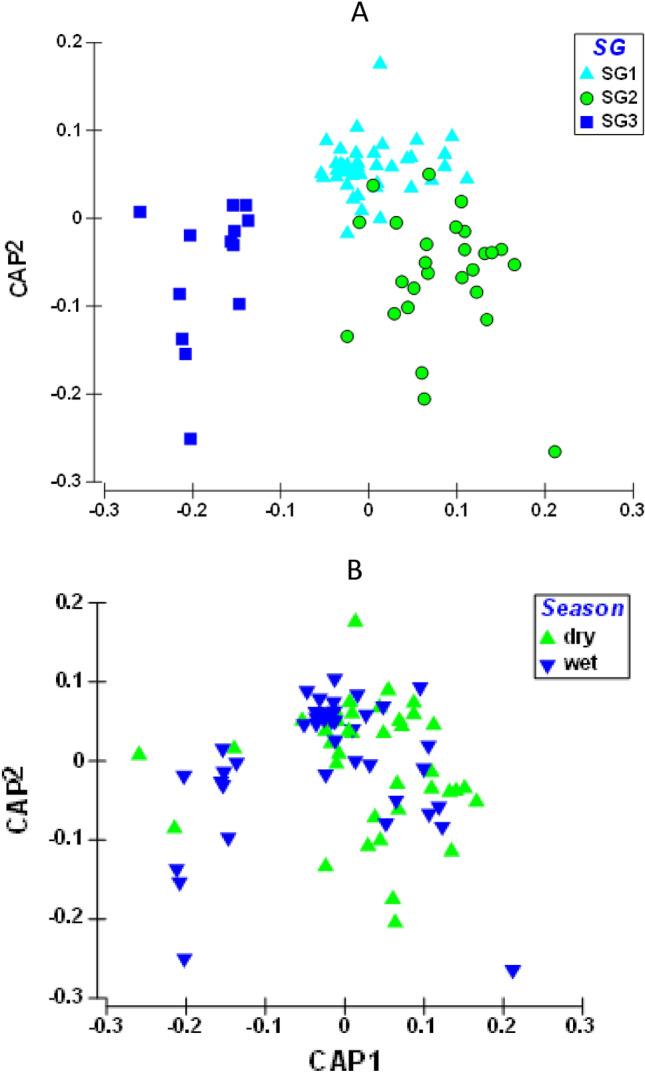
Canonical analysis of principal coordinates (CAP) ordination plot showing the clustering of site groups (**A**) and seasons (**B**) based on the physicochemical variables during the study period (2008–2012). *SG1* Site group 1 (Site 1 = Adofi, Site 2 = Oleri, and Site 3 = Anwai upstream, Site 4 = Anwai downstream and Site 9 = Ogba upstream site), *SG2* Site group 2 (Site 5 = Ethiope upstream, Site 6 = Ethiope downstream, Site 7 = Obosh and Site 8 = Ogba downstream) > *SG3* Site group 3 (Site 10 = Orogodo and Site 11 = Warri River).

The CAP revealed physicochemical variables were different among the three sites groups (Fig. 1). The percent site classification into parent groups was 8% (Tables 1, 2). The least impacted Site group was separated from other sites towards axis 2 of the CAP plot, supporting our site classification (Fig. 2). Site groups 1 and 2 were also clearly separated, but not as much as those of Site group 3. PERMANOVA showed that both land-use and season significant influenced water quality variables in our study, however the effect of urban land use was higher than that of season (Site group: F = 42.288, *p* = 0.001; Season: F = 3.287; *p* = 0.06; Table 2). However, the interaction between site groups and season had a significant effect on water quality in the study area (F = 6.7429; *p* = 0.009).

**Table 1:** Summary results of Canonical analysis of principal coordinates (CAP) analysis for average differences in physicochemical variables among the 11 sites in the three site groups.

Original group	SG1	SG2	SG3	Total	%correct	Total %correct
SG1	43	1	0	44	97.73	79/84 (94%)
SG2	4	23	0	27	85.19	
SG3	1	0	144	13	100

**Table 2:** PERMANOVA result showing the significant differences in physicochemical variables among the three site groups in the Niger Delta Region Streams during the study period (2008–2012).

Source	df	SS	MS	Pseudo-F	P(perm)	perms
SG	2	10,974	5487.1	42.288	0.001	998
Se	1	426.52	426.52	3.287	0.06	998
SGxSe	2	1749.9	874.94	6.7429	0.009	998
Res	78	10,121	129.76			
Total	83	22,066				

			Pairwise			
**Groups**						
SG1, SG2				8.7368	0.001	997
SG1, SG3				2.2984	0.017	999
SG2, SG3				4.5187	0.002	998

*SG* site group, *Se* season, *Res* residuals.

### Macroinvertebrate functional composition

**Figure 3:**
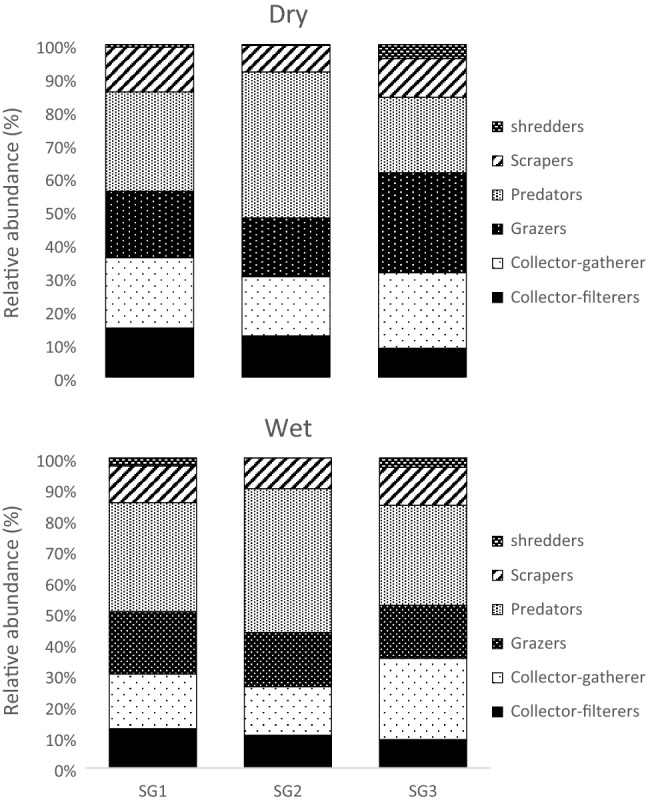
Relative abundance of macroinvertebrate functional feeding groups (FFGs) across the 11 river stations in three site categories Site group 1 (Site 1 = Adofi, Site 2 = Oleri, and Site 3 = Anwai upstream, Site 4 = Anwai downstream and Site 9 = Ogba upstream site), SG2: Site group 2 (Site 5 = Ethiope upstream, Site 6 = Ethiope downstream, Site 7 = Obosh and Site 8 = Ogba downstream) > SG3: Site group 3 (Site 10 = Orogodo and Site 11 = Warri River) during the dry and wet seasons.

The most common FFG in the Niger Delta Streams sites sampled at both seasons were the predators, accounting for 32.12% and 38.25% of all FFGs collected during the dry and wet seasons, respectively (Table 3). This was followed by collector-gatherers and grazers that recorded over 40.0% each for both seasons (Table 3). The least abundant FFG was the shredders, which accounted for 1.77% and 1.077% during the dry and wet seasons, respectively (Table 3). Generally, predators increased with increasing urban pollution gradient, with their lowest relative abundance values recorded in the least impacted Site group 3 at both seasons (Table 3, Fig. 3). Shredders declined with increasing urban pollution at both season (Fig. 3). PERMANOVA showed that the effect of land use significantly affected the relative abundance of FFGs (F = 5.820; *p* = 0.001), but season had no effects on the relative abundance of FFGs (F = 0.263; *p* = 0.878 Table 4).

**Table 3:** Relative abundance (RA) of functional feeding groups (FFGs) of macroinvertebrates across the sampling site categories analysed in this study during the dry and wet seasons (2008–2012).

SG	Collector-filterers	Collector-gatherer	Grazers	Predators	Scrapers	Shredders
**Dry**						
SG1	14.63	20.99	19.81	29.60	13.34	0.75
SG2	12.41	17.74	17.60	43.72	7.95	0.27
SG3	8.79	23.08	30.40	23.08	11.72	4.29
Total	35.84	61.80	67.81	96.40	33.01	5.32
**Wet**						
SG1	12.63	17.65	20.13	35.11	11.81	0.75
SG2	10.57	15.63	17.45	46.35	9.96	0.38
SG3	9.14	26.23	17.09	32.32	12.18	2.06
Total	32.34	59.50	54.67	113.78	33.95	3.19

SG1 (Site group 1), SG2 (Site group 2) and SG3 (Site group 3).

**Table 4:** PERMANOVA result showing the significant differences in the relative abundance of macroinvertebrate functional feeding groups variables among the three site groups in the Niger Delta Region Streams during the study period (2008–2012).

PERMANOVA table of results						
Source	df	SS	MS	Pseudo-F	P(perm)	Unique perms
SG	2	16,966.000	8483.100	5.820	0.001	997.000
Se	1	383.250	383.250	0.263	0.878	999.000
SGxSe	2	2372.800	1186.400	0.814	0.559	997.000
Res	78	113,690.000	1457.600			
Total	83	133,640.000				

*SG* site group, *Se* season, *Res* residuals.

### Relationships between environmental variables and macroinvertebrate FFGs

**Figure 4:**
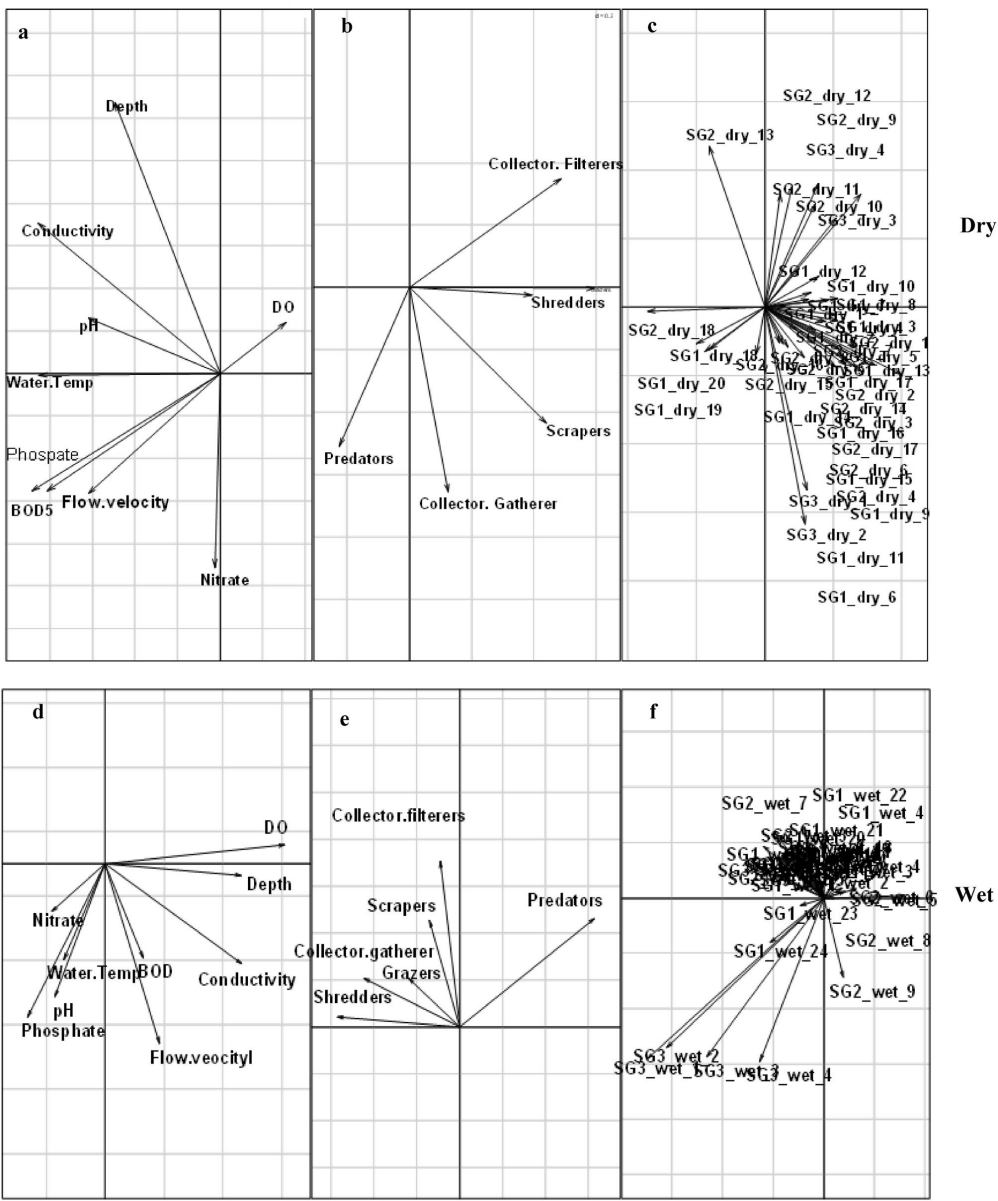
RLQ analysis showing the site grouping during the study period based on the analysed physicochemical variables (**a**,**d**), and the distribution of FFG (**b**,**e**) in relation to the sampling sites (**c**,**f**) in three station categories three site categories: Site group 1 (Site 1 = Adofi, Site 2 = Oleri, and Site 3 = Anwai upstream, Site 4 = Anwai downstream and Site 9 = Ogba upstream site), SG2: Site group 2 (Site 5 = Ethiope upstream, Site 6 = Ethiope downstream, Site 7 = Obosh and Site 8 = Ogba downstream) > SG3: Site group 3 (Site 10 = Orogodo and Site 11 = Warri River).

A total of 52 macroinvertebrate families were identified and assigned to the six FFGs across the 11 sites in three site groups, during the dry and wet seasons (see Supplementary Information, Table S1). During the dry period, the RLQ analysis revealed that axes 1 and 2 with eigenvalues of 0.21 and 0.0061, respectively, explained a cumulative variance of 93% of the total variance in the RLQ model, indicating that the two axes accounted for most of the variability in the analysis (Table 5). In the wet season, axes 1 and 2 with eigenvalues 0.11 and 0.01, respectively explained 92%. Generally, the RLQ ordination model revealed that FFGs responded differentially to increasing urban pollution between the site groups. Except for few FFGs such as predators, most FFGs exhibited consistent responses between the both dry and wet seasons in the study area (Fig. 4). For example, predators, collector-gatherers, scrapers, and shredders were negatively associated with EC and pH at the moderately impacted sites during the dry season (Sites 5, 7 and 8; Fig. 4). Similarly, these FFGs, alongside grazers and collector-filterers indicated negative associations with EC, including BOD_5_, flow velocity and increased water depth at the moderately impacted sites (Fig. 4). On the other hand, predatory macroinvertebrates showed strong positive associations with nutrients (phosphate and nitrates), water temperature and BOD_5_ during the dry seasons, indicating strong positive relationship with Site group 1. At both seasons, collector-filterers were sensitive, indicating strong negative correlations with majority of water quality variables, including water temperature, nutrients and BOD_5_ and EC (Fig. 4).

**Table 5:** Properties of the RLQ analysis for the FFGs, environment and taxa datasets collected in the Niger Delta Streams during the study period (2008–2012).

Properties	Dry		Wet	
	Axis 1	Axis 2	Axis 1	Axis 2
Variance	71.74	20.99	77.52	13.98
Cumulative variance (%)	71.74	92.73	77.52	91.5
Eigenvalues	0.21	0.061	0.11	0.01
Correlation	1.54	0.86	1.29	1.49

## Discussion

In Nigeria, water resource managers are presented with the overwhelming task of developing effective biomonitoring tools to assess and manage pollution from urban activities, including industrialisation and oil exploration^9,43,46^. In this study, we explored the community patterns of macroinvertebrate FFGs to urban pollution, hoping to identify indicator FFGs for long-term urban pollution monitoring in the Niger Delta Region of Nigeria. Our physicochemical results showed a strong correlation between nitrate and BOD_5_ with highly impacted site groups, indicating that urban land use played a significant role in our study’s water quality and habitat characteristics. Seasonality did not show a marked influence on urban disturbance effects on water quality, with physicochemical indicators of urban land use not differing between the dry and wet seasons (Table 2).

The increased nutrient concentrations in the highly disturbed sites are consistent with previous studies in urban impacted areas in the tropics^10,29,66^. Similarly, earlier studies^9,46,67–69^ reported a significant increase in nutrient enrichment in the Niger Delta Regions of Nigeria. Urban activities in the regions are associated with poorly managed sewage and industrial facilities^67,70^ and limited access to good water delivery services, causing nutrient loading through erosion and runoffs from the surrounding catchment. This problem may suggest increased nutrient concentrations, including electrical conductivity observed in our study's highly impacted and moderately impacted site groups. Pollution in the area has also been attributed to the location of an abattoir upstream of site 10 (Orogodo River), and the presence of an extensive oil exploration company in site 11 (Warri River). The Niger Delta Region is an oil-rich region and thus an economic hub and hosts to most of the crude oil production activities from upstream to downstream^71,72^, with severe implications on river pollution through oil spillage. Therefore, it can be inferred from the physiochemical variable result that most of the sampling sites in the present study area are subjected to a high degree of anthropogenic influences affecting aquatic communities.

Predators dominated the relative abundance of macroinvertebrate FFGs in the study area, whereas shredders were the least represented FFGs. Shredders (Trichoptera and Potamanthidae) are more sensitive to environmental changes, while collectors and predators are more tolerant to disturbance and organic pollution^35,73^. For example, previous studies have observed predators be sensitive to pollution via biomagnification of metals and dissolved ions and impaired feeding efficiency due to visual impairment from suspended sediments^74,75^. However, predators such as hemipterans and coleopterans possess specialised physiological structures such as breathing tubes, which enables them to breathe atmospherically in the face of increasing pollution gradient^10,26,29^. Thus, although predators are specialist macroinvertebrate feeders likely to be vulnerable to urban pollution, the possession of air breathing structures by most of them may have confer resilience, explaining their increased abundance in impacted sites at both seasons. Further, macroinvertebrate predator abundance is influenced by the availability of prey animals^10,26^. In our study, such tolerant prey animals such as the oligochaetes were dominant in the disturbed sites, suggesting the increased abundance of predators in urban impacted sites. Thus, the strong positive correlation of predators and shredders with electrical conductivity and water depth on the RLQ model suggest predators and shredders are probably indicators of dissolved ions caused by runoff from farmlands and urban areas^76,77^. Nevertheless, the response patterns of predators and shredders in our study area may also indicate intermediate pollution from urban disturbance, as predators were mainly associated with the moderately impacted site groups and showed close associations with DO during the wet period. Similar studies have reported the dominance of predators in sites within urban centers^10,26,39^. Similarly, the paucity of shredders is consistent with findings from urban land use impacted sites in the tropic (e.g.^36,41,42^).

Gatherers are macroinvertebrates that feed on coarse particulate organic matters (CPOM^31^). Gatherers were predominant in Sites 7 and 8, the moderately impacted site group, and on the RLQ ordination model, they were strongly associated with depth and electrical conductivity (Fig. 3). Considering that gatherers rely on CPOM for their food, their preponderance in the moderately impacted site group was as expected as they rely on the quality availability of food. Earlier studies had emphasised the importance of CPOM to the survival of gatherers in aquatic systems^24^. Removing riparian vegetation (e.g., for agricultural purposes) and industrial advancement reduces allochthonous inputs to rivers^78^, resulting in decreased food availability for gatherers. Further, most of the gatherers in our study are sensitive to clogging by sediments, and urban activities increase fine sediment into rivers systems^79^. The sensitivity of collectors-gatherers have been reported in urban impacted river systems in temperate streams^80,81^. Similarly, the dominance and sensitivity of gatherers in our study have been reported in riverine systems in tropical streams^29,41,42,82,83^ and their dominance is predicated on the fast decline of forested dominated sites in rivers in the tropics most especially Africa where population explosion is a serious concern to forested riverine systems.

On the other hand, shredders, grazers, and scrapers increased with urban pollution and were identified as tolerant to urban pollution. Grazers were associated with BOD_5_ and nitrate on the RLQ ordination model, while scrapers were associated with electrical conductivity and water depth. Grazing and scraping macroinvertebrates feed on algal and macrophytes; hence their association with BOD_5_ and nitrate is not unexpected. Fertilizer runoff entering a stream enhances the development of periphytic algae, thus favouring grazer-scrapers^84,85^. Increased nutrient enrichment can stimulate the growth of macrophytes and algal, increasing food availability for grazing and scraping macroinvertebrates, suggesting the increased abundance of grazers and scrapers in polluted sites in our study area. Further, removing riparian vegetation in streams and rivers catchments for agricultural and urban settlement purposes result in increased light exposure that effectively promotes primary algal production^78^, ultimately favouring the occurrence of grazing and scraping macroinvertebrates. The increased light exposure in urban impacted sites may probably be attributed to the significant positive correlation of grazers to impacted sites in the RLQ model  (Fig. 4). Similarly, a study in an industrial and urban dominated river in South Africa was reported to be dominated by grazers and they were found to be sensitive to pollution^10^. Although, it was termed surprising by the researcher, as grazers are supposed to be increase with increasing nutrients and other pollution indicating environmental variables such as BOD_5_ and electrical conductivity. Our results which showed scrapers to be associated with electrical conductivity is as expected as studies in the tropics have reported the predominance of scrapers in rivers and streams subjected to anthropogenic influences^19,83^.

Shredders are essential food guild in an aquatic food chain^29^. However, in the present study, shredders were the least abundant FFGs. This result agrees with other studies in the tropics^86,87^. Since most shredders are pollution sensitive taxa (e.g., Trichoptera, Ephemeroptera and Plecoptera (EPT), their low abundance is not surprising. Taxa in the aquatic insect group of EPT have consistently been reported to be sensitive to environmental disturbances^73,88^. Earlier studies have shown that in tropical streams and rivers, the abundance of shredders varies temporally due to the influence of agricultural and urban sites resulting from deforestation^86,87^. These findings are similar to our findings regarding the once forested catchments streams and rivers in the Niger Delta Region of Nigeria which have been replaced with urban and agricultural catchments^86,87^. However, contrary to other studies in the tropics, we found shredders tolerant of urban pollution in our research. Shredders indicated positive correlations with the moderately impacted sites and increasing water depth on the RLQ model (Fig. 4). Temperature and nutrients are often the primary factors determining litter decomposition rates^89,90^. In our study, a possible explanation for the tolerance of shredders to urban pollution may be attributed to the stimulated microbial decomposition of leaves and algae in the study area, providing more quality and palatable food for shredding macroinvertebrates. The stimulating effects of temperature and nutrients on leaf litter decomposition have been demonstrated in streams and river systems^89,91^. Further, in this study, we employed multivariate analysis of individual FFG to environmental indicators of urban pollution, and such approach is susceptible to interactive effects of other FFGs (see^10,91–94^). Thus, shredders responses to urban disturbance may have been mediated by the interactive effects of other FFGs. Several riverine studies have reported the interactive effects of response variables on communities' responses to pollution (e.g.^95–97^). We recommend further studies in other riverine systems to better understand the sensitivity of Afrotropical macroinvertebrate FFGs.

## Conclusion and recommendation

Our study revealed that the functional organisations of macroinvertebrates in the Niger Delta Region are differentially affected by urban pollution. Predators, scrapers/grazers and shredders were classified as tolerant to urban pollution, whereas collector-filters were sensitive to urban disturbance. Although studies have found that macroinvertebrate FFGs are influenced by land uses, but proper attention is not paid to identifying specific FFG indicators of environmental disturbance, including urban pollution. Thus, this study contributes significantly by identifying potentially sensitive FFGs of urban pollution from an Afrotropical river system. We, therefore, hope that through further research, such FFG indicators can be sufficiently used for long-term monitoring like taxonomical indicators where specific metrics such as EPT abundance and diversity have been established through decades of research as being sensitive to environmental disturbance, including urban pollution. We, therefore, recommend further studies on FFGs response to environmental disturbance in the Afrotropical stream to confirm the sensitivities of macroinvertebrate FFGs to urban pollution.

## Supplementary Information

Supplementary Figure S1.

Supplementary Table S1.

## Data Availability

Datasets used in the study is available at Dryad at 10.5061/dryad.98sf7m0dq.
