# Supplementary material for: Insights from the Niger Delta Region, Nigeria on the impacts of urban pollution on the functional organisation of Afrotropical macroinvertebrates
Source: Sci Rep. 2022 Dec 29;12:22551. doi: 10.1038/s41598-022-26659-0 (PMC9800367; doi:10.1038/s41598-022-26659-0)
Supplement: Supplementary file 1 — Supplementary Figure S1. [file 41598_2022_26659_MOESM1_ESM.docx]

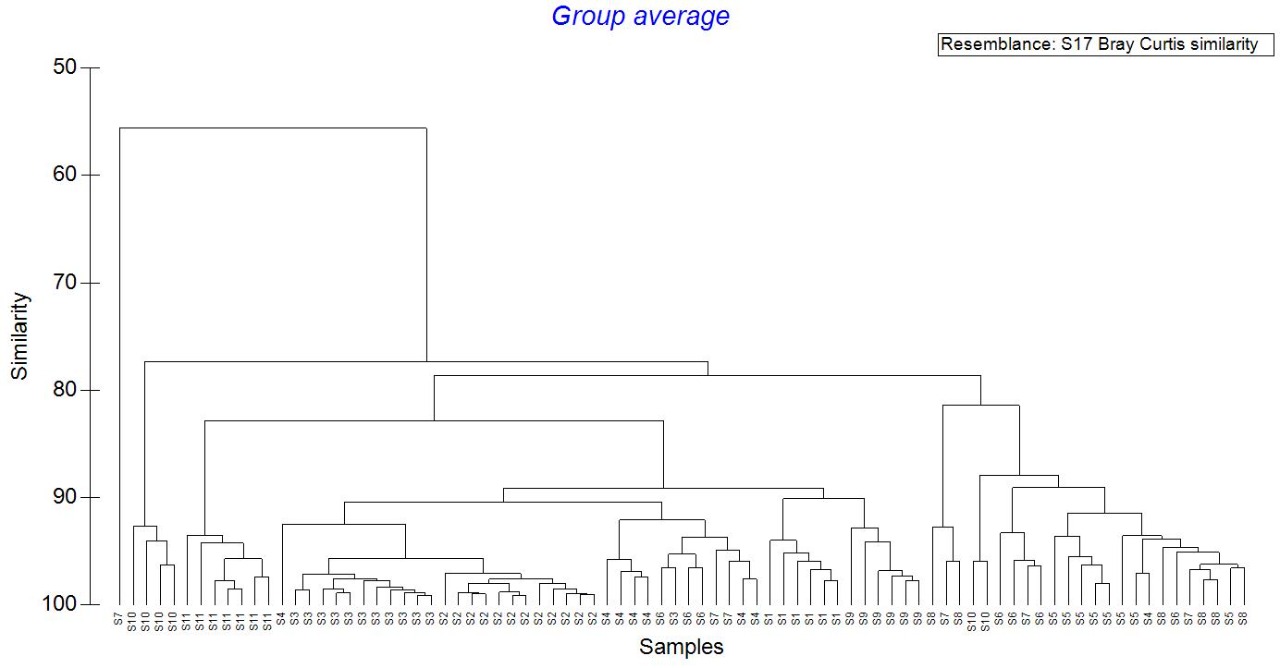


**Figure S1:** Cluster analysis of sites classification based on physicochemical variables collected from the 11 sites across the eight streams in the Niger Delta Region, Nigeria. **Note:** Site group 1 (Sites 1, Site 2, Site 3, Site 4 and Site 9 = Ogba upstream site); Site group 2 (Sites 5, Site 6, Site 7 and Site 8); Site group 3 (Sites 10 and Site 11).
