# Supplementary material for: Insights from the Niger Delta Region, Nigeria on the impacts of urban pollution on the functional organisation of Afrotropical macroinvertebrates
Source: Sci Rep. 2022 Dec 29;12:22551. doi: 10.1038/s41598-022-26659-0 (PMC9800367; doi:10.1038/s41598-022-26659-0)
Supplement: Supplementary file 2 — Supplementary Table S1. [file 41598_2022_26659_MOESM2_ESM.docx]

**Supplementary Table S1:** Binary coding of functional feeding groups (FFGs) in urban stream sites in the Niger Delta Region of Nigeria

| **Taxa** | **Functional feeding groups (FFGs)** | | | | | |
| --- | --- | --- | --- | --- | --- | --- |
|  | Predators | Scrapers | Grazers | Collector-filterers | Collector-gatherer | Shredders |
| Naididae | 0 | 0 | 1 | 0 | 1 | 0 |
| Tubificidae | 0 | 0 | 0 | 0 | 1 | 0 |
| Lumbricidae | 0 | 0 | 0 | 0 | 1 | 0 |
| Lymnaidae | 0 | 1 | 1 | 0 | 0 | 0 |
| Planorbidae | 0 | 0 | 1 | 0 | 0 | 0 |
| Thiaridae | 0 | 0 | 1 | 0 | 0 | 0 |
| Amphullariidae | 0 | 0 | 1 | 0 | 0 | 0 |
| Atyidae | 0 | 0 | 0 | 0 | 1 | 0 |
| Desmocarididae | 1 | 0 | 1 | 0 | 0 | 0 |
| Euryrhynchidae | 1 | 0 | 1 | 0 | 0 | 0 |
| Palaemonidae | 0 | 0 | 0 | 0 | 1 | 0 |
| Baetidae | 1 | 1 | 1 | 1 | 1 | 0 |
| Leptophlebiidae | 0 | 0 | 0 | 0 | 1 | 0 |
| Caenidae | 0 | 0 | 0 | 0 | 1 | 0 |
| Heptageniidae | 0 | 0 | 1 | 0 | 0 | 0 |
| Tricorythidae | 0 | 0 | 0 | 0 | 1 | 0 |
| Oligoneuridae | 0 | 0 | 0 | 0 | 1 | 0 |
| Perlidae | 1 | 0 | 1 | 0 | 1 | 0 |
| Hydroptilidae | 0 | 0 | 0 | 0 | 1 | 0 |
| Hydropsychidae | 1 | 0 | 0 | 0 | 0 | 0 |
| Ecnomidae | 0 | 0 | 0 | 0 | 1 | 0 |
| Leptoceridae | 1 | 1 | 1 | 1 | 1 | 1 |
| Pyraustidae | 0 | 0 | 1 | 0 | 0 | 0 |
| Notonectidae | 1 | 0 | 0 | 0 | 0 | 0 |
| Pleidae | 0 | 1 | 0 | 1 | 0 | 0 |
| Mesoviliidae | 1 | 0 | 0 | 0 | 0 | 0 |
| Nepidae | 1 | 0 | 0 | 0 | 0 | 0 |
| Naucoridae | 1 | 0 | 0 | 0 | 0 | 0 |
| Belostomatidae | 1 | 0 | 0 | 0 | 0 | 0 |
| Gerridae | 1 | 0 | 0 | 0 | 0 | 0 |
| Dytiscidae | 1 | 0 | 0 | 0 | 0 | 0 |
| Hydrophilidae_ | 1 | 0 | 0 | 0 | 0 | 0 |
| Elmidae | 0 | 1 | 0 | 0 | 1 | 1 |
| Gyrinidae | 1 | 0 | 0 | 0 | 0 | 0 |
| Noteridae | 1 | 0 | 0 | 0 | 0 | 0 |
| Hydraenidae | 0 | 1 | 0 | 0 | 1 | 1 |
| Aeschnidae | 1 | 0 | 0 | 0 | 0 | 0 |
| Gomphidae | 1 | 0 | 0 | 0 | 0 | 0 |
| Coenagrionidae | 1 | 0 | 0 | 0 | 0 | 0 |
| Libellulidae | 1 | 0 | 0 | 0 | 0 | 0 |
| Calopterygidae | 1 | 0 | 0 | 0 | 0 | 0 |
| Macromidae | 1 | 0 | 0 | 0 | 0 | 0 |
| Chlorocyphidae | 1 | 0 | 0 | 0 | 0 | 0 |
| Culicidae | 0 | 0 | 0 | 1 | 0 | 0 |
| Simulidae | 0 | 0 | 0 | 1 | 0 | 0 |
| Tabanidae | 0 | 0 | 0 | 1 | 0 | 0 |
| Ceratopogonidae | 0 | 0 | 1 | 0 | 0 | 0 |
| Athericidae | 1 | 0 | 0 | 0 | 0 | 0 |
| Chaoboridae | 1 | 0 | 0 | 0 | 0 | 0 |
| Tipulidae | 1 | 0 | 0 | 0 | 0 | 0 |
| Sryphidae | 1 | 0 | 0 | 1 | 0 | 0 |
| Chironomidae | 1 | 0 | 0 | 0 | 0 | 0 |
